# Antioxidant Activity of Bovine Colostrum in the Colon of a Mouse Model of TNBS-Induced Colitis

**DOI:** 10.3390/antiox14020232

**Published:** 2025-02-18

**Authors:** Leonardo Leonardi, Shadi Dib, Egidia Costanzi, Gabriele Brecchia, Giovanna Traina

**Affiliations:** 1 Department of Veterinary Medicine, University of Perugia, 06126 Perugia, Italy; leonardo.leonardi@unipg.it (L.L.); egidia.costanzi@unipg.it (E.C.); 2 Department of Pharmaceutical Sciences, University of Perugia, 06126 Perugia, Italy; shadideeb_2006@yahoo.com; 3 Department of Veterinary Medicine and Animal Sciences, University of Milan, 26900 Lodi, Italy; gabriele.brecchia@unimi.it

**Keywords:** colitis model, TNBS, bovine colostrum, antioxidant expression, catalase, glutathione peroxidase, superoxide dismutase

## Abstract

(1) Background: Bovine colostrum (BC) is the initial milk produced by cows after giving birth and has revealed significant potential in helping various health conditions, particularly in diseases of the gastrointestinal tract, such as inflammatory bowel disease, including colitis. BC is renowned for its rich composition of components that strengthen the immune system. Inflammatory bowel diseases, including colitis, are characterized by elevated oxidative stress, leading to tissue damage and exacerbated symptoms. The aim of this study was to explore the potential antioxidant activity of bovine colostrum in the context of a mouse model of trinitrobenzene sulfonic acid-induced colitis. The effectiveness of BC in mitigating oxidative stress and its effects on colitis was evaluated. (2) Methods: Mice were divided into two groups, one group received BC by gavage for 21 days, the other group received saline solution; after 21 days one half of each of the two groups of mice were treated intrarectally with trinitrobenzene sulfonic acid to induce colitis. Colon samples were processed by immunocytochemical methods. The immunoreactivity of the main antioxidant enzymes, (i) catalase (CAT), (ii) superoxide dismutase 1 (SOD1), (iii) superoxide dismutase 2 (SOD2) and glutathione peroxidase 4 (GPX4), at the colon level was analyzed. (3) Results: The results showed positive immunoreactivity of catalase and SOD2 activities of BC in the colon of animals after induction of inflammation. (4) Conclusions: The findings have the potential to suggest new strategies for the management of gastrointestinal disorders related to oxidative stress. Furthermore, the knowledge gained could contribute to the development of functional foods or supplements specifically designed for the management of colitis. Future studies will be aimed at identifying the bioactive fractions of BC to study the mechanisms underlying its actions, as well as to trace which populations can benefit most from colostrum consumption, in addition to subjects with gastrointestinal disorders.

## 1. Introduction

Colostrum, also called “the first food of life”, is the milk produced by mammals in the days immediately following birth—generally around 3 or 4 days—before its transformation into mature milk [1,2]. Bovine colostrum (BC) shares some similarities with human colostrum while showing distinct composition variations; BC is notable for its higher concentrations of immunoglobulins, IgG and IgM, in contrast to human colostrum, which displays elevated levels of constituents such as lactose, lactoferrin and IgA [3,4,5,6,7]. Both forms of colostrum play a crucial role in supporting the growth of newborns thanks to their abundant reserves of carbohydrates, proteins, fats, vitamins and minerals [5,6,7].

Colostrum also provides a shield against pathogens, facilitates the maturation of the immune system and promotes a harmonious balance of intestinal microorganisms. Colostrum includes growth factors that contribute to the development and repair of several tissues, including the intestinal lining [4]. Colostrum contains a significantly higher concentration of immunostimulatory compounds and growth factors than mature milk and many components, enzymatic and non-enzymatic, with potential antioxidant activity [5,6,7,8,9,10]. Colostrum is a rich source of compounds with antioxidant activity, including carotene, lycopene, retinol, tocopherol, lactoperoxidase, catalase (CAT), superoxide dismutase (SOD), glutathione peroxidase (GPx), lactoferrin, vitamins A, E, C, selenium, copper and zinc [3,4,5,8].

Previous studies have also demonstrated the anti-inflammatory effect of BC using an experimental animal model of intestinal inflammation [11,12]. Inflammation and oxidative stress are strongly interconnected. Oxidative stress and inflammation reinforce each other by positive feedback, extending the inflammatory response that when overactivated causes damage to the host [13]. On the other hand, the generation of reactive oxygen species (ROS) can be stimulated by inflammatory processes. High oxidative stress can trigger and exacerbate inflammatory reactions and, therefore, inflammatory state. Studies have highlighted the crucial role of oxidative stress in the development and progression of inflammatory bowel diseases (IBDs), as well as in ROS production and antioxidant defense systems [13,14]. Oxidative stress is an imbalance between the generation of ROS and antioxidant defense mechanisms in the body, resulting from excessive ROS generation or a deficiency of antioxidant systems [13]. An excessive generation of ROS or a deficiency of antioxidant systems leads to the disruption of redox regulation and possible damage to cellular components such as lipids, DNA, proteins and carbohydrates [14]. ROS molecules have been shown to activate the gene expression of molecules involved in promoting inflammation, ultimately leading to a state of chronic inflammation in the body. Among the various disorders affected by oxidative stress, inflammatory bowel disease (IBD) has emerged as a key focus of research due to the complex relationship between the imbalance of ROS and antioxidant defense systems in the gastrointestinal tract [14]. Studies have revealed that murine colitis models have shown an increase in the ratio of ROS/reactive nitrogen species (RNS) [15]. The insufficiency of antioxidant enzymes, such as catalase (CAT), glutathione peroxidase (GPx) and superoxide dismutase (SOD), has been noticed in the colonic mucosa, submucosa and serosa of IBD patients compared to the small intestine [16]. Furthermore, non-enzymatic antioxidants, including vitamins, carotene, tocopherol and glutathione, are reduced [15,16]. As a result, oxidative stress plays a crucial role in the development and worsening of IBD, and is not just a consequence of ongoing inflammation. This could be attributed to a combination of genetic predisposition and environmental factors. However, there are various substances, like antioxidants, hormones, synthetic compounds, polyphenols, herbal extracts, specific foods, essential nutrients and probiotics, that show promise as potential treatments for reducing oxidative stress in IBD [17,18,19,20,21,22,23]. More research is necessary to verify if combining these new therapies that enhance antioxidants with standard medications can enhance the overall treatment of IBD.

Many treatments involving antioxidants reduce oxidative damage while alleviating intestinal inflammation. The prospect of oxidative stress in colitis means that antioxidant therapy could be a potential strategy for the management and treatment of IBD [17,18,19,20,21,22,23].

The aim of this study was to explore the potential antioxidant activity of BC in the inflammatory context of a mouse model of trinitrobenzene sulfonic acid (TNBS)-induced colitis. The effectiveness of BC in mitigating oxidative stress and its effects on colitis were evaluated. The immunoreactivity of the main antioxidant enzymes, (i) catalase (CAT), (ii) superoxide dismutase 1 (SOD1), (iii) superoxide dismutase 2 (SOD2) and glutathione peroxidase 4 (GPX4), was analyzed at the colon level in mice treated for 21 days with BC. 

## 2. Materials and Methods

### 2.1. Animal

Six-week-old CD-1 male mice (30.1 ± 1.41 g) were used, obtained from Harlan Laboratories S.r.l. (Correzzana D’Adda, Milan, Italy), and housed in controlled conditions at a temperature of 21 ± 1 °C, relative humidity of 55 ± 10% and 12 h light/dark cycles. After an acclimatization period of 10 days, during which the mice were provided with water and food ad libitum, they were included in the experimental trial. At the end of the trial, the mice were euthanized, and their tissues were collected for further analysis. The study had official approval from the Ethical Committee for Animal Experimentation at the University of Perugia, Italy. All experimental protocols in this study adhered to Italian regulations (Ministerial Declaration 116/92) and European Economic Community regulations (O.J. of the European Commission L 358/1 18 December 1986). The care of animals followed guidelines to minimize distress, and only the essential number of animals were used to ensure reliable outcomes. The study defined a humane endpoint as a loss of >20% of body weight; however, no mice met this criterion.

### 2.2. Bovine Colostrum

We used Nutra Summa Pure Bovine Colostrum Powder^®^ (Phoenix, AZ, USA), which was skimmed, pasteurized and freeze-dried to ensure minimum denaturation of Ig and of the other bioactive molecules. The nutrition information of the BC used is Protein 46 g, Total Fat 24 g, Carbohydrates 20 g, Sugars 20 g and Sodium 0.4 g.

### 2.3. Study Groups

**Figure 1:**
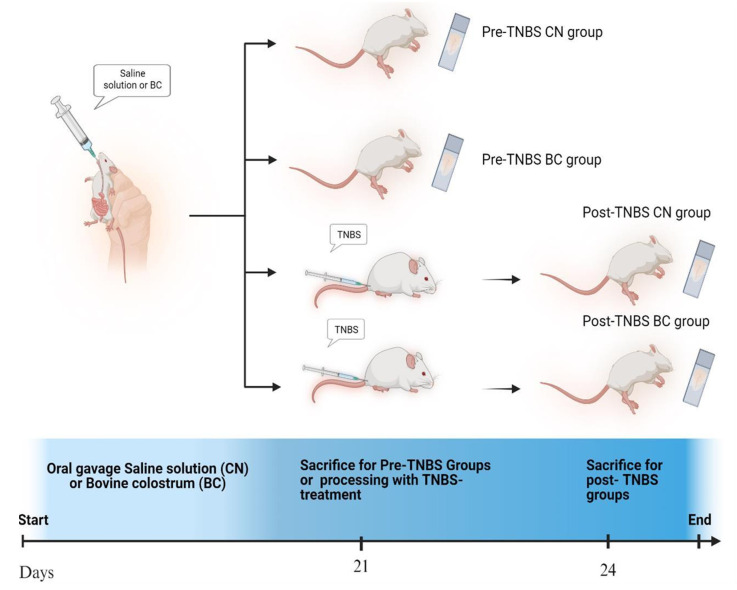
Overview of the study groups (Created in BioRender.com).

Figure 1 shows the experimental protocol as reported in Filipescu et al. [12]. Firstly, CD-1 mice (n = 24) were randomly divided into two groups (n = 12).

Mice of the control group (CN) were subjected to daily oral administration of saline solution, whereas those in the BC group received an equivalent volume of bovine colostrum dissolved in saline solution for 21 days. 100 mg of colostrum was dissolved in 0.6 mL of saline solution for each mouse in BC group or the same volume of saline solution in control group, according to previous studies [9,12]. The delivery of solutions was accomplished via oral gavage, directly into the stomach. Following the 21-day treatment duration, a portion of the mice were sacrificed for analysis, while the remaining mice were utilized for colitis-induction through TNBS (Figure 1). To induce colitis, the mice were first fasted for 24 h, according to the procedure reported in [17,18]. Following this fasting period, mice were lightly anesthetized with isoflurane. Subsequently, 150 μL of a 1% TNBS solution, prepared by diluting the sensitizing agent in 50% ethanol at the time of use, was injected into the rectum of the mice through a plastic catheter equipped with a 1 mL syringe (Figure 1). To ensure uniform distribution of the TNBS throughout the colon for effective colitis induction, the mice were kept in a head-down position for some minutes [19,20].

Three days post-TNBS induction, these mice were also sacrificed for further investigation (Figure 1).

### 2.4. Tissue Processing

Mice were euthanized using cervical dislocation. Each mouse was then thoroughly examined in a clean and controlled setting, beneath a fume hood, to ensure that the samples were not contaminated. The entire digestive tract, starting at the esophagus and ending at the anus, was carefully and hygienically removed. Subsequently, the colon was separated and opened lengthwise under sterile conditions. The contents within the colon were carefully collected and preserved in sterile, pre-reduced phosphate-buffered saline (PBS). These samples were immediately frozen at a temperature of −80 °C for later microbiological and molecular analysis. After cleaning, colon tissue sections were placed in a 10% neutral buffered formalin solution for fixation. The tissue then underwent a series of steps, including dehydration using ascending concentrations of ethanol, clearing in xylene and paraffin embedding at a controlled temperature. Once embedded, thin sections (approximately 4–5 μm) were cut using a microtome and transferred to a water bath to expand them. The sections were then mounted on glass slides for immunohistochemistry and histology analysis.

### 2.5. Primary Antibodies

#### 2.5.1. Anti-Catalase Antibody

The rabbit monoclonal antibody, Anti-Catalase antibody (EPR1928Y), sourced from Abcam (Cambridge, UK), was used at a 1:100 dilution for immunohistochemistry on paraffin-embedded tissue. Antigen retrieval employed heat treatment with tris-EDTA buffer at pH 9 prior to staining.

#### 2.5.2. Anti-Superoxide Dismutase 1 Antibody

The rabbit monoclonal antibody SOD1 (E4G1H) XP^®^ Rabbit mAb, sourced from Cell Signaling Technology (Danvers, MA, USA) was used at a 1:250 dilution for immunohistochemistry on paraffin-embedded tissue. Antigen retrieval employed heat treatment with tris-EDTA buffer at pH 9 prior to staining.

#### 2.5.3. Anti-Superoxide Dismutase 2 Antibody

The rabbit monoclonal antibody SOD2 (D3 × 8F) XP Rabbit mAb, sourced from Cell Signaling Technology, was used at a 1:250 dilution for immunohistochemistry on paraffin-embedded tissue. Antigen retrieval employed heat treatment with tris-EDTA buffer at pH 9 prior to staining.

#### 2.5.4. Anti-Glutathione Peroxidase 4 Antiboby

The rabbit polyclonal antibody GPX4 (#52455), sourced from Cell Signaling Technology, was used at 1:250 dilution for immunohistochemistry on paraffin-embedded tissue.

### 2.6. Immunohistochemistry Protocol

The sections were first deparaffinized in xylene for 20 min, then in decreasing alcohol concentrations of 95%, 80%, 70%, 50% and finally in distilled water. Following this, antigen retrieval was performed using a pre-heated Tris-EDTA pH 9 buffer in a microwave for 2 min at maximum power. The sections were then placed in this pre-heated buffer and subjected to microwave heating for 20 min at minimum power. Afterward, they were allowed to cool down for 20 min. The sections were initially washed in a TBS–Tween solution, followed by a 10 min incubation in a 3% H_2_O_2_ solution to block endogenous peroxidase activity. After rinsing in TBS–Tween again, barriers were drawn around the tissue using a pap pen to restrict reagent flow. Subsequently, the sections were placed in a wet box, and a 10 min application of Protein blocking buffer (ab93677, Abcam) was employed to block proteins. Primary antibodies (50–70 μL), previously diluted as specified, were then applied and incubated for 2 h. After further washing in TBS–Tween, a 10 min application of the secondary antibody (biotinylated link ab93677, Abcam) was followed by streptavidin incubation (ab93677, Abcam), each with subsequent TBS–Tween washes. AEC chromogen (ab64252, Abcam) was used for approximately 10 min, followed by rinsing in distilled water and a 5 min differentiation in hematoxylin. The sections were then washed with tap water, mounted and covered with an aqueous riser (Aquatex, Merck KGaA, Darmstadt, Germany). The intensity of immunolabeling was qualitatively evaluated by two investigators at different times with light microscopy Olympus BX51, camera Olympus DP70 (Olympus, Segrate, Italy). The immunoreactivities were considered negative, weak, moderate or strong.

### 2.7. Statistical Analysis

The body weight (BW) of individual mice was measured daily. As reported in detail in Filipescu et al. [12], changes over time (before and after TNBS treatment) and between groups were assessed using linear mixed models. The models evaluated the effect of time, group and their interaction. Sidak correction was applied for multiple comparisons. Statistical significance was set at *p* < 0.05. Statistical analyses were performed with SPSS Statistics version 23 (IBM, SPSS Inc., Chicago, IL, USA).

## 3. Results

### 3.1. Macroscopic Evaluation

The colitis symptoms and histological lesions of the colon as well as the weight of the animals in the various experimental groups were evaluated according to Filipescu et al. [12]. Firstly, in the macroscopic examination, the colon of the BC pre-TNBS mice and the control group did not show any lesions; on the contrary, the colon of the post-TNBS control mice appeared swollen, edematous and thickened, and showed evidence of mucosal hemorrhage compared to the BC post-TNBS group. The administration of BC was very well tolerated, and did not induce any pathological symptoms.

Before TNBS treatment, the body weight (BW) of all mice increased from day 2 to 21 (33.2 ± 0.1 g and 34.5 ± 0.1 g at day 2 and 21, respectively, *p* < 0.001) without differences between groups (34.8 ± 0.1 g and 35.0 ± 0.1 g in CN and BC groups, respectively, *p* = 0.442). After TNBS treatment, the BW of the BC group was higher than the CN group (32.5 ± 0.4 g and 35.4 ± 0.5 g in CN post-TNBS and BC post-TNBS groups, respectively, *p* = 0.003).

### 3.2. Catalase Expression

**Figure 2:**
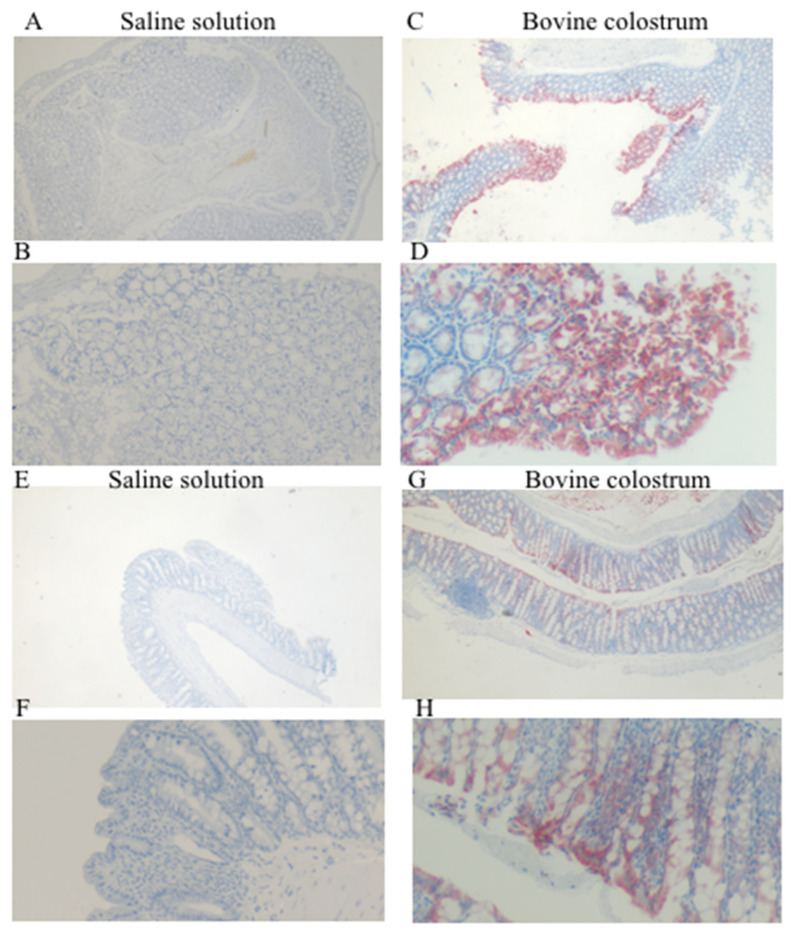
Immunohistochemical staining for anti-catalase, with (**A**,**B**) representing the saline control group and (**C**,**D**) the BC treatment group, each shown at magnifications of 4× and 20×, respectively. Immunohistochemical staining for anti-catalase, with (**E**,**F**) representing the saline control post-TNBS group, and (**G**,**H**) the BC post-TNBS group, each shown at magnifications of 4× and 20×, respectively.

Before TNBS treatment, the saline solution group exhibited negative staining, whereas the BC group displayed strong positive staining, particularly at the mucosal level of the colon. Following TNBS treatment, the saline solution group continued to exhibit negative staining, while the BC group showed a moderate staining pattern within the mucosa (Figure 2).

### 3.3. Superoxide Dismutase 1 Expression

**Figure 3:**
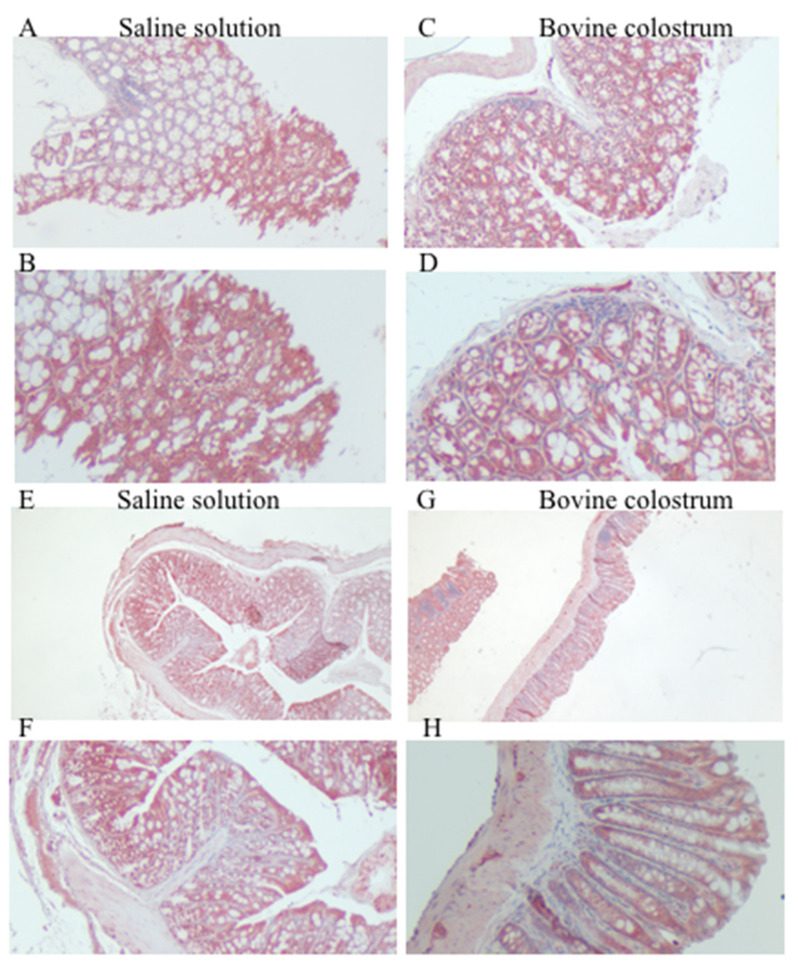
Immunohistochemical staining for anti-SOD1, with (**A**,**B**) representing the saline control group and (**C**,**D**) the BC treatment group, each shown at magnifications of 4× and 20×, respectively. Immunohistochemical staining for anti-SOD1, with (**E**,**F**) representing the saline control post-TNBS group, and (**G**,**H**) the BC post-TNBS group, each shown at magnifications of 4× and 20×, respectively.

Before TNBS treatment, both the saline solution group and the BC group exhibited strong positive staining, with no significant differences between the two groups. After TNBS treatment, the staining pattern remained consistently strong and positive, and there were no significant differences observed between the groups (Figure 3).

### 3.4. Superoxide Dismutase 2 Expression

**Figure 4:**
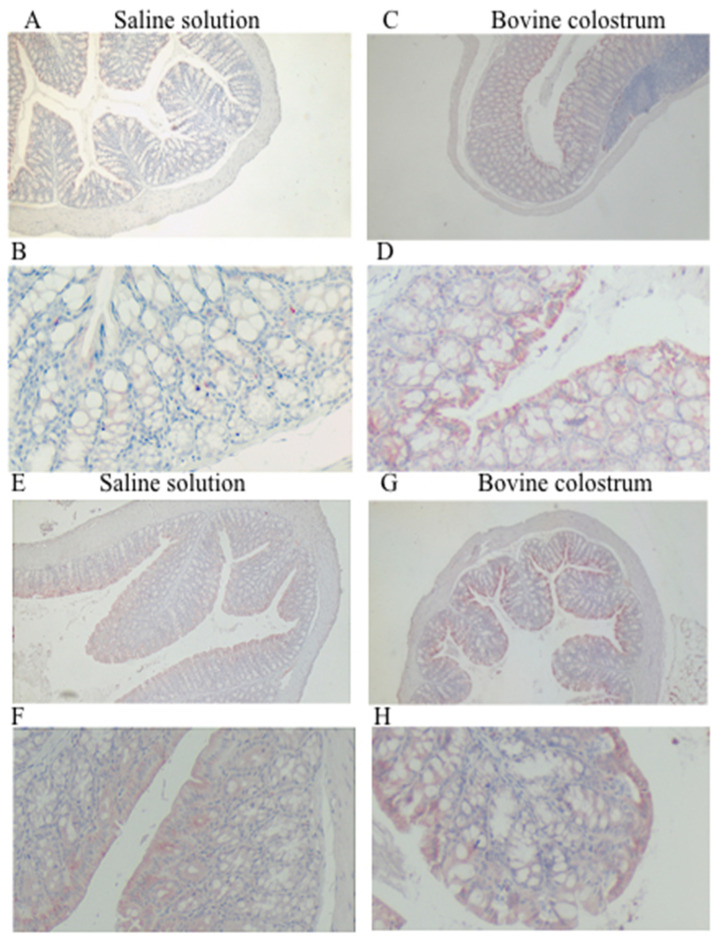
Immunohistochemical staining for anti-SOD2, with (**A**,**B**) representing the saline control group and (**C**,**D**) the BC treatment group, each shown at magnifications of 4× and 20×, respectively. Immunohistochemical staining for anti-SOD2, with (**E**,**F**) representing the saline control post-TNBS group, and (**G**,**H**) the BC post-TNBS group, each shown at magnifications of 4× and 20×, respectively.

Before TNBS treatment, staining showed moderate levels for both the saline solution group and the BC group, with no differences observed between the two groups. However, following TNBS treatment, the BC group exhibited a moderate staining pattern, while the saline solution group displayed weaker staining. This difference was notable, especially in the context of the mucosal level of the colon (Figure 4).

### 3.5. Glutathione Peroxidase 4 (GPX4) Expression

**Figure 5:**
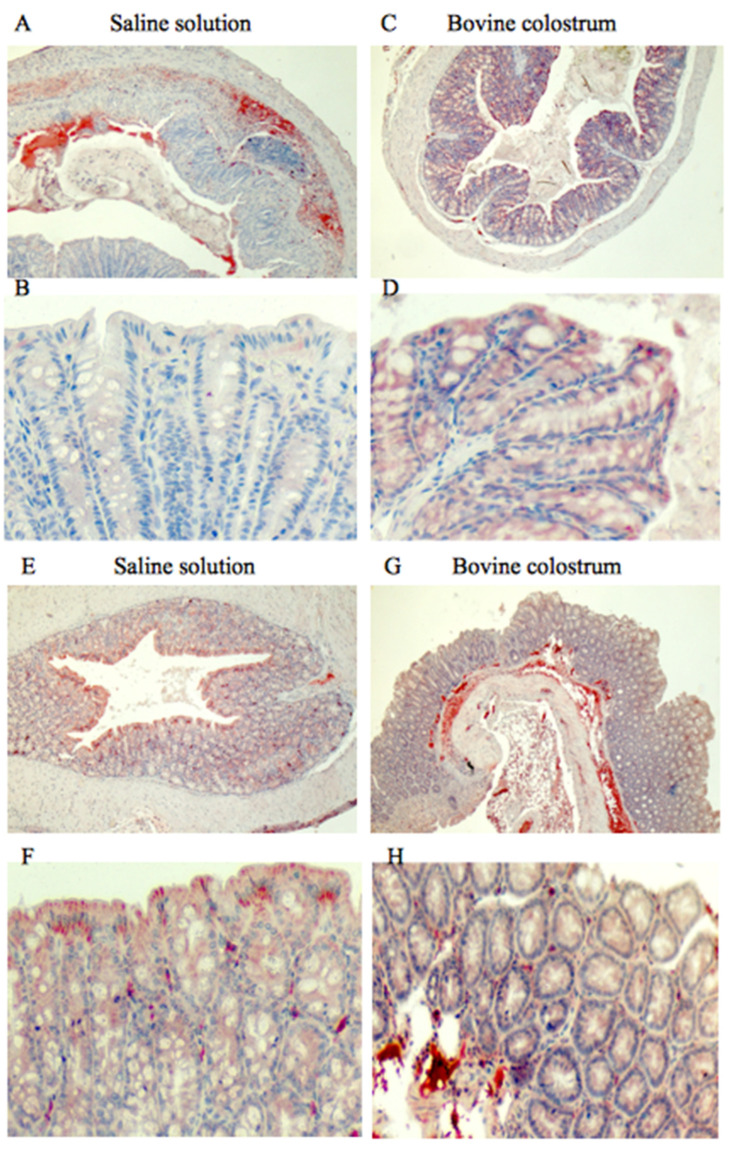
Immunohistochemical staining for anti-GPx4, with (**A**,**B**) representing the saline control group and (**C**,**D**) the BC treatment group, each shown at magnifications of 4× and 20×, respectively. Immunohistochemical staining for anti-GPx4, with (**E**,**F**) representing the saline control post-TNBS group, and (**G**,**H**) the BC post-TNBS group, each shown at magnifications of 4× and 20×, respectively.

The immunohistochemistry of colons from the control mice demonstrated GPx4 expression in all cells of the intestinal epithelium, including enterocytes, goblet cells and neuroendocrine cells. GPx4 immunoreactivity was present in both the nucleus and cytoplasm (Figure 5).

## 4. Discussion

The relationship between the dietary intake of antioxidant/bioactive compounds and their health effects has long been an important question. IBD, including CD and UC, is characterized by chronic inflammation in the gastrointestinal tract, with contributions from immune system dysfunction, genetics and environmental factors [23]. A promising avenue is suggested using colostrum as an adjuvant or alternative therapy [3,4,5,6,7,8,9,12].

BC is an attractive candidate due to its rich composition, which includes nearly 90 bioactive compounds, such as immunoglobulins, growth factors, antibodies, amino acids, oligosaccharides, antibacterial compounds and immunological regulators. Compared to human colostrum, BC is 100–1000 times more potent in providing passive immunity and the growth factors essential for physical and gastrointestinal development. Furthermore, these compounds of considerable interest maintain their biological efficacy as they pass through the gastrointestinal tract, resulting in a positive impact on intestinal function [24,25]. In addition, colostrum is a rich source of compounds with potential antioxidant activity [3,4,5,8].

BC showed the potential to modulate the immunological response as well as the severity of the intestinal inflammatory reaction modulating Toll-Like Receptor 4 and cytokine expression, reducing body weight loss and histological score, balancing the microbiota and finally decreasing the clinical signs of colitis in mice [12].

The present study aimed to investigate whether BC possesses antioxidant activity in an in vivo colitis model, and to verify whether the anti-inflammatory action that it has demonstrated in colitis conditions [9,12] accompanies the antioxidant response. Thus, we assessed the main antioxidant enzymes (SOD1, SOD2, CAT, GPx) in a TNBS-induced colitis model in animals previously treated with BC. These enzymes are considered the first line of antioxidant defense and are significant in the prevention of oxidative damage [13,14,15,16,23].

The results presented report positive immunoreactivity of the CAT, GPx and SOD2 activities of BC in animal colons after the induction of inflammation. Overall, our results confirm previous observations [25], showing a greater protective effect of BC. An imbalance between ROS generation and antioxidant defense mechanisms disrupts redox regulation and can damage cellular components such as lipids, DNA, proteins and carbohydrates [26]. Oxidative stress is crucial for the development and worsening of IBD, influenced by genetic predisposition and environmental factors. In addition, oxidative stress is a response of the body stimulated by TNBS [27]. Previous studies have demonstrated a notable increase in ROS levels in mouse models of colitis, underscoring their role in the pathogenesis of IBD [28]. Oxidative damage is related to the severity of the disease and is also present in significant quantities in the normal-appearing mucosa of colitis subjects, suggesting that oxidative damage does not inevitably lead to tissue damage and is not a consequence of tissue damage [29].

An essential factor contributing to the development of IBD is an impaired antioxidant defense system [30]. CAT, SOD and GPX are all essential enzymes that constitute endogenous antioxidant enzymes [31]. CAT is a crucial enzyme that catalyzes the decomposition of hydrogen peroxide into water and oxygen, thus protecting cells from oxidative damage [32]. In some cases, where CAT is absent its functions can be performed by glutathione peroxidase. CAT can also act in the so-called peroxidative mode, where its functions involve the decomposition of small substrates such as methanol. Another important function of CAT is apoptosis [33,34]. Altered CAT activity is observed in subjects with colitis, suggesting an imbalance in antioxidant defenses [25]. In colitis conditions, CAT prevents loss in ion transport [34].

It has been suggested that decreased CAT activity is an important indicator of the antioxidant insufficiency of defense mechanisms, possibly resulting from enzymatic damage or the exhaustion of the catalase processing capacity itself [30,31].

In the present study, we have demonstrated that there is a significant increase in CAT staining in the BC-treated group compared to the CN-treated group. CAT immunoreactivity is also evident in the post-TNBS group. This higher staining observed in the BC group suggests a potential improvement in antioxidant defense mechanisms in the colitis model. Further studies may evaluate the ability of BC to re-establish physiological transport phenomena across the intestinal barrier.

SOD is an antioxidant enzyme that catalyzes the conversion of superoxide radicals into oxygen and hydrogen peroxide [32]. It was observed that a deficiency of the SOD1 enzyme exacerbated inflammation and resulted in reduced activity of the antioxidant enzyme, thus confirming its importance [35]. SOD is a primary catalyst that regulates RNS and ROS by directly associating with superoxide. It is therefore an essential signaling molecule. Studies report that in mice with colitis SOD2 is downregulated [36]. In the present study there was no significant difference in SOD1 expression between the CN and BC groups, neither before nor after TNBS treatment. It is possible that SOD1 may already be maximally expressed in response to induced colitis, leaving little room for further potentiation by BC. Further investigations, such as gene expression analysis, could shed light on the mechanisms underlying SOD1 regulation in this context. In contrast to SOD1, SOD2 expression was higher in the BC-treated group. SOD2, located in the mitochondria, plays a fundamental role in the neutralization of superoxide radicals generated during oxidative metabolism. Increased SOD2 staining in the BC group suggests the potential mitigation of mitochondrial oxidative stress. Among BC components with antioxidant activity, it has been reported that lactoferrin, a multifunctional iron-binding glycoprotein with a major protective role, can itself increase endogenous antioxidant mechanisms [36]. Our findings suggest that BC may establish a functioning antioxidant defense mechanism. This result is consistent with previous research that has shown that BC supplementation can increase SOD activity [37,38,39,40].

The antioxidant action of BC could probably be exerted through its role on the integrity of the epithelial barrier [41,42], but also on the action that BC exerts on the muco-microbial layer [12]. In this regard, our previous study examined the impact of BC on intestinal microbiota. TNBS treatment led to a smaller increase in *E. coli* levels in the BC group than in the CN group, potentially mitigating its adverse effects, while beneficial bacterial strains, such as *Enterococci*, *Lactobacillus* spp. and *Bifidobacterium* spp., were higher in mice that received BC [12]. Such findings also confirmed the role of BC in promoting the growth of beneficial intestinal bacteria, potentially contributing to improved gut health [9,12].

This study contributes to the growing body of evidence supporting the potential benefits of BC in mitigating the oxidative stress associated with colitis. The mechanisms underlying the observed effects of BC on CAT and SOD2 staining remain to be elucidated. One hypothesis is that BC contains many bioactive components and enzymatic and non-enzymatic antioxidants that could directly or indirectly affect the expression or activity of these enzymes. Future studies should explore these potential mechanisms, including the identification of specific bioactive components in BC that may modulate antioxidant defenses. Understanding the precise mechanisms through which BC exerts its antioxidant effects is critical to the development of targeted therapies and dietary interventions for patients suffering from these conditions.

However, it is important to note that BC can also be a source of ROS, since its constituent lipids are sensitive to the peroxidation process and contain xanthine oxidase and lactoperoxidase, which are ROS-generating enzymes [11]. The main function of GPx is to reduce hydroperoxide levels [43,44,45]. GPx4 was detected in several cell organelles, including cytosol, mitochondria, nucleus and endoplasmic reticulum (ER) [44]. GPx4 activity has been detected in the colons of mice. Among glutathione peroxidases, GPx4 stands out for its broad substrate specificity and its protective function in a variety of cellular compartments. GPx4 prevents cell membrane damage and subsequent cell death, due to its ability to reduce phospholipid hydroperoxides and repress lipid peroxidation derived from 12/15-lipoxygenase [45]. Furthermore, a role for GPx4 in preventing mutagenesis has been suggested. In the murine intestine, GPx4 mRNA is one of the most abundant selenoprotein transcripts [46].

A major advantage of the colitis model employed in this study is the exceptional reproducibility of TNBS use, which ensures consistent and reliable results across different experiments. Furthermore, TNBS demonstrates notable specificity in inducing inflammation primarily at the administration site in the colon. This targeted inflammatory response allows researchers to focus their investigations on the unique reactions of the colonic mucosa to insult [47]. The impact of BC on TNBS-induced colitis using this TNBS model supports the preventive effect of the colostrum on inflammation [9,12]. The study demonstrates that pretreatment with BC in the TNBS group resulted in milder architectural changes and a less severe inflammatory response with more organized crypts; in contrast, severe architectural changes were observed in the CN group after TNBS treatment. Importantly, BC treatment was well tolerated and did not induce side effects. Furthermore, in a previous study [12], an increased expression of TLR4, IL-1β and IL-8 was observed in the colon following TNBS treatment. Mice in the BC group showed lower levels of these inflammatory markers than the CN group after TNBS treatment. Preclinical studies support the administration of antioxidants with anti-inflammatory activity in the treatment of IBD [48,49]. This confirms that BC may have a protective, preventive effect by reducing inflammation in the colon in response to TNBS-induced colitis. The present study highlights the antioxidant activity of BC in a TNBS model.

The strength of this study is that it allowed us to detect the presence of enzymatic immunoreactivity with antioxidant activity in the tissues of animals with colitis induced after having previously taken BC. A limitation of the experimental approach used is that it is fundamentally a qualitative analysis, and the actual amount of antioxidant activity cannot be defined in an absolute way. Therefore, the results provide an important indicator for further molecular investigation.

## 5. Conclusions

Bovine colostrum is a heterogeneous complex of multiple biologically active compounds, making it a precious natural substance. The present study revealed interesting antioxidant properties of BC in a model of intestinal inflammation and confirmed them through the immunohistochemical analysis of CAT, GPx and SOD2. An aspect that makes BC even more attractive is its excellent tolerability profile, with rare side effects. Furthermore, its affordability and availability position it as a potentially promising future option for managing various health conditions, including IBD. However, it is essential to highlight that further research is imperative to fully understand this relationship and its underlying mechanisms. Such study will have the potential to expand our knowledge and may also reveal the effects of BC not only on IBD but also on other diseases. This opens interesting possibilities for the broader application of BC as a therapeutic agent in various medical contexts and general health.

Due to its characteristics and functional activities, BC has the potential to be developed both as a functional food and to enhance the activity of foods and/or their potential health benefits. Future studies will be aimed at identifying the bioactive fractions of BC to study the mechanisms underlying its actions, as well as to trace which populations can benefit most from colostrum consumption, in addition to subjects with gastrointestinal disorders.

## Data Availability

The data supporting the results are available from the offices of Professors Leonardo Leonardi and Giovanna Traina.
